# Healthy Bread

**Published:** 1877-07

**Authors:** 


### HEALTHY BREAD

Is advertised by some man in Boston, who has discovered a *Dietetic Saleratus*, which he avers to possess two remarkable characteristics.

1st. It is *pure* saleratus which "is not *so* destructive to the digestive organs as the common kind," thus admitting the "destructive qualities" of both!

2nd. This saleratus makes eight pounds of bread out of seven pounds of flour, intimating that there is one-eighth more of nutriment in a pound of flour made into bread with his saleratus, than there was in the flour itself, which is a manifest untruth; the weight is increased, but the amount of nutriment is not. Does successful trade require such deceptions? We think not.

Saleratus will always increase the evil which it is designed to remedy, because, to produce a specified effect, the quantity must be increased from time to time, or it will become wholly inefficient.

In the second place, there is nothing curative in saleratus; it removes a present effect, which we call "*acidity*," but it has no possible tendency to prevent that acidity the very next time food is eaten. Acidity is caused by want of power in the stomach to digest that kind of food which, when eaten, gives rise to what is commonly called *Heart-burn*. To prevent this acidity, we must give the stomach more power, more strength to perform its accustomed—its natural work.

Now, when a man feels weak, what does he do? He rests; rest gives strength; and until his strength improves, he does such light work as that strength will allow. So to give more power to the stomach, we must give it more rest by giving it less work to do, which is done in two ways.

*First.* By eating less frequently, we allow more time for repose, for recuperation. A dyspeptic person is hungry all the time; is eating all the time; because, if he does not, he "*feels bad;*" but if he yields to his appetite, he very soon feels worse; it is only for the few minutes his food is passing down his throat that he feels better, for which he pays hours of subsequent suffering. This is a specimen of man's wisdom.

*Second.* To afford rest for the stomach, we must give it easy work, by studying and observing what kinds of food it manages most readily, but that great simpleton, man, is too lazy or too ignorant to make such observations, and goes at once to taking some kind of "Bitters" or "Tonic," with the avowed object of "improving his appetite;" that is to say, to enable him to eat more, when the fact is, he is already eating too much! In addition to that, instead of observing what his own stomach receives most kindly, he turns round and begins to eat what somebody else said cured him. To make your field produce largely of a certain crop, you must put upon it that kind of manure which contains most largely that element which the field lacks, and which that particular crop most requires. To make the stomach yield the most nutriment to the body, you must put into it that kind of food which has the element which the particular body most requires; nature—instinct—tells what that is. We know it is said by some that the appetite is vitiated, and is not a safe guide, but this is rarely so; the error is, not that what nature calls for is unwisely called for, but that man is such a glutton, he cannot be satisfied with a moderate quantity of what is called for, but must stuff himself like a pig, must gobble it down until he can scarcely swallow another particle. Shame on our want of rationality and self-denial! In *dyspepsia, acidity, heart-burn, indigestion*, or by whatever name it may be called, our highest wisdom consists in the first place in giving preference to that kind of food which nature most craves, and if, after several trials, that food is invariably followed by some discomfort, we must not conclude that such food, although strongly craved, is unsuitable of itself; it is safer to infer that mischief has resulted, not from the quality of the food, but from its quantity; and very certainly we will arrive at the quantity which will not only give no discomfort, but which may be eaten with satisfaction, and disposed of without an effort, and that article should be continued as an aliment until nature takes a dislike to it and craves something else. These are the principles which should guide us in the class of ailments we have named, and there is more virtue in them than in all medicine; but happily for us doctors, the people have neither the intelligence to perceive their wisdom, nor the firmness to carry them out.

We ought to know that our Maker is beneficent enough to cause that kind of food to flourish most in the locality where the human residents most need its elements. How strange that infatuation which causes us constantly to overlook the multitudes of evidences about us of the forethought of our Creator! This great principle is evidenced in our finding meats and animal oils almost exclusively as the aliment of the Greenlander, while fruits in rich profusion are found in all tropical countries, fruits being cooling, and meats and oils necessary to keep up an internal fire where quicksilver freezes.

On this self-evident principle we found our conclusion, that, had it been better for us to have had saleratus in our corn and wheat, the Almighty would have placed them there in such combination as no Bostonian could ever hope to equal.

If a man's stomach is healthy and strong, he needs no saleratus in his bread: if it is not healthy and strong, bread will sour, if largely eaten, because the stomach can digest only a small quantity of it. If saleratus is put in the bread, more bread can be eaten without souring on the stomach, but no power is added to the stomach to digest that larger quantity; the saleratus has kept under a single effect, that is, souring or fermentation, and the ability of digestion not being increased, we only, by the use of saleratus, give the stomach a greater work to perform, and keep it longer at it, when we should have given it a less task, thus enabling it to get through its work the sooner, and consequently have a longer time for recuperation. Surely it requires no great depth of thought to see into these things.

